# Interpersonal Conflict and Employee Behavior in the Public Sector: Investigating the Role of Workplace Ostracism and Supervisors’ Active Empathic Listening

**DOI:** 10.3390/bs15020194

**Published:** 2025-02-12

**Authors:** Hatem Belgasm, Ahmad Alzubi, Kolawole Iyiola, Amir Khadem

**Affiliations:** Department of Business Administration, Institute of Graduate Research and Studies, University of Mediterranean Karpasia, 33010 Mersin, Turkeykolawole.iyiola@akun.edu.tr (K.I.);

**Keywords:** interpersonal conflict, workplace ostracism, interpersonal deviance, supervisors’ active empathic listening, stressor–emotion model, conservation of resources theory, conflict expression framework

## Abstract

In today’s dynamic organizational environments, interpersonal conflict and social exclusion can significantly impact employee behavior and organizational effectiveness. This study explores the complex interplay between interpersonal conflict, workplace ostracism, and interpersonal deviance in Jordan’s public sector, emphasizing the moderating role of supervisors’ active empathic listening. Using the stressor–emotion model, conservation of resources (COR) theory, and conflict expression (CE) framework, this study examined these relationships through a two-wave survey design. Data were collected from 501 public sector employees using validated scales, and an analysis was conducted using SPSS and AMOS, with structural equation modeling employed for hypothesis testing. The findings reveal that interpersonal conflict strongly predicts workplace ostracism and interpersonal deviance. Workplace ostracism mediates the relationship between conflict and deviance, while supervisors’ active empathic listening moderates these effects, reducing the likelihood of deviant behaviors. These results underscore the importance of fostering empathetic leadership and inclusive workplace environments to mitigate conflict’s negative impact. This research contributes to understanding workplace dynamics by highlighting the critical role of supervisors in moderating conflict and ostracism. The findings have practical implications for public sector organizations. Beyond training programs, supervisors can implement active empathic listening in practical settings by regularly holding one-on-one meetings in which they actively listen to employee concerns, using verbal and non-verbal cues to show engagement, asking open-ended questions to encourage deeper discussion, reflecting employee emotions to validate their feelings, and following up on issues raised to demonstrate concrete action based on what they have heard; this creates a culture of open communication in which employees feel heard and valued, leading to increased employee engagement and improved problem-solving abilities.

## 1. Introduction

The workplace is a social environment where employees engage in various interactions that contribute to their productivity, satisfaction, and overall well-being (Xiao et al., 2020). In any organization, interpersonal conflict is inevitable and can arise from misunderstandings, competition for resources, or differing values. Defined as tension or discord between employees, interpersonal conflict can manifest in various forms, such as arguments, disagreements, or passive resistance (Saepulloh & Laksana, 2022; Zhao et al., 2020). While some conflicts can foster creative problem-solving and innovation, prolonged or unresolved interpersonal conflict often leads to negative outcomes (Engelbrecht, 2023). Additionally, emotions play a significant role in workplace conflict, often acting as triggers for disputes and influencing how individuals react to disagreements, with supervisors’ emotional management being particularly crucial, as their behavior can greatly impact team dynamics and conflict resolution; negative emotions like anger, frustration, or stress can escalate conflicts, while positive emotions can foster collaboration and constructive problem-solving.

A crucial consequence of interpersonal conflict is interpersonal deviance, which highlights actions that not only breach organizational norms but also threaten the well-being of colleagues. It is essential to recognize the impact these behaviors have on the workplace environment (Naz & Siddiqui, 2021). Interpersonal deviance includes actions such as gossiping, undermining colleagues, or withholding important information, which can negatively affect team cohesion and overall organizational effectiveness (Ogunfowora et al., 2021). 

In the public sector, which is often characterized by rigid hierarchies, formal communication channels, and organizational processes, the effects of interpersonal conflict can be even more pronounced. Public sector dynamics refers to the complex interplay of forces within government agencies and institutions that influence how they operate, make decisions, and deliver services to citizens; its relevance lies in its direct impact on a nation’s economic development, social welfare, and overall quality of life by shaping policies related to crucial areas like healthcare, education, infrastructure, and others, ensuring that essential services are provided to the public, even in areas that the private sector might not reach. Employees working in government or public institutions frequently face stressors such as limited resources, political pressures, and a high degree of accountability to the public. 

In such an environment, interpersonal conflict can aggravate stress and lead to behaviors that undermine workplace coordination (Palancı et al., 2020).

Workplace ostracism encapsulates the distressing experience of feeling ignored, excluded, or isolated by colleagues or supervisors, a situation that can significantly impact morale and productivity (Zahid et al., 2021). Ostracism is a form of social exclusion that can have severe psychological and behavioral consequences for employees. Studies have shown that workplace ostracism can lead to reduced job satisfaction, emotional withdrawal, and even increased turnover intention (Fatima et al., 2023). Moreover, ostracized employees may resort to deviant behaviors to manage their exclusion or to react against their peers (Gamian-Wilk & Madeja-Bien, 2021). 

Researchers have found that active empathic listening, in which supervisors listen attentively, understand employees’ emotions, and respond with empathy, improves workplace relationships and lessens the impact of stressors like conflict and ostracism. By providing emotional support and facilitating communication, supervisors who engage in active empathic listening can prevent conflicts from escalating and reduce the likelihood of deviant behaviors (Manusov et al., 2020).

This study draws on three key theoretical frameworks, the stressor–emotion model, the conservation of resources theory, and the conflict expression framework, to investigate the effect of workplace ostracism on employee behavior because these theories help explain the relationship between interpersonal conflict, workplace ostracism, and interpersonal deviance in the public sector. In reducing increases in psychological stress, reluctance to share knowledge, declining job satisfaction, and workplace deviant behaviors, the stressor–emotion model (Spector & Fox, 2005) is a simplified representation of these phenomena. The COR theory is a set of established principles that attempt to explain and predict a phenomenon (Hobfoll, 1989), and the CE framework (Todorova et al., 2022) aims to explain a phenomenon by providing a set of interconnected concepts and principles. The stressor–emotion model, the conservation of resources theory, and the conflict expression framework based on a cohesive narrative are all theoretical models used to understand and analyze how stressors and conflicts impact individuals and groups. In addition, they approach the issue from different angles, with the stressor–emotion model focusing on the direct link between stressors and emotions, the conservation of resources theory looking at how stressors deplete valuable resources, and the conflict expression framework examining how shared narratives shape conflict dynamics. The stressor–emotion model explains how workplace stressors, such as interpersonal conflict, can lead to emotional responses that drive deviant behaviors (Zahid & Nauman, 2024). 

The COR theory underscores the inherent human drive to acquire and protect essential resources, including social connections and emotional health. We can foster deeper relationships and enhance our overall well-being by valuing these aspects. Threats to these resources can trigger significant stress and lead to deviant behavior (Wang et al., 2023). Meanwhile, the CE framework addresses the dynamics of conflict communication and management in the workplace, emphasizing how ostracism can profoundly affect conflict outcomes. Understanding these theories can enhance workplace harmony and well-being (Weingart et al., 2015). These theoretical perspectives offer a comprehensive view of how interpersonal conflict can lead to deviance through social and emotional mechanisms.

Interpersonal conflict is a source of employee behavior that has an impact on organizational outcomes (Ahmad, 2022). Along with organizational outcomes, interpersonal conflict can facilitate workplace ostracism (Brougham & Haar, 2020). This study aims to fill the existing gaps by examining the potential mediating effect of workplace ostracism on the connection between interpersonal conflict and interpersonal deviance. This exploration could enhance our understanding and contribute to the field significantly. Addressing this gap is critical for advancing our understanding of these dynamics. Despite its recognition as a significant form of social exclusion, the role of workplace ostracism as a mediator in this research remains unfamiliar. 

Research demonstrates that supervisors’ behaviors significantly affect employees’ responses to conflict. However, there remains a gap in understanding how active empathic listening specifically moderates the relationship between interpersonal conflict and workplace ostracism and their impact on deviant behavior. Addressing this gap could lead to more effective strategies for managing workplace dynamics. While the private sector dominates much of the existing literature on workplace deviance, it is crucial to acknowledge the dearth of research on the public sector. The organizational structures and stressors in public organizations are unique and can significantly influence workplace behavior, making it crucial to explore these dynamics further. Given the existing research gaps, this study is poised to explore critical questions that can enhance the understanding of workplace dynamics:

1. In which ways does interpersonal conflict impact interpersonal deviance among public sector employees?
2. How does workplace ostracism serve as a mediator in the relationship between interpersonal conflict and interpersonal deviance?
3. What is the moderating effect of supervisors’ active empathic listening on the relationship between workplace ostracism and interpersonal deviance?
4. How does supervisors’ active empathic listening influence the indirect relationship between interpersonal conflict and deviance through workplace ostracism?
5. How do the stressor–emotion model, of resources theory, and conflict expression framework help in promoting employee behavior in the public sector?

The core objectives of this research are critical for advancing our understanding of public sector dynamics. This research aims to delve into the complex interplay between interpersonal conflict and interpersonal deviance in the public sector, to analyze the role of workplace ostracism as a mediator in the interaction between interpersonal conflict and deviance, to evaluate how supervisors’ active empathic listening serves as a moderator in the relationship between workplace ostracism and interpersonal deviance, And to investigate how supervisors’ active empathic listening affects the indirect influence of interpersonal conflict on deviance through workplace ostracism.

We meticulously organize this paper into several impactful sections. Section 1 begins with a compelling introduction that not only presents essential concepts, but also identifies critical research gaps, formulates thought-provoking questions, defines clear objectives, and outlines the significant contributions of the study. Section 2 delivers a thorough literature review, critically analyzing previous studies on interpersonal conflict, workplace ostracism, interpersonal deviance, and active empathic listening, and also examining the robust theoretical frameworks that underlie this research. Section 3 details a well-structured methodology, covering the research design, effective data collection procedures, and analytical techniques employed to rigorously test the hypotheses. Section 4 showcases the results of the statistical analyses, offering strong evidence in support of the hypothesized relationships. Section 5 engages in a thoughtful discussion of the findings, skilfully connecting them to the theoretical frameworks and drawing valuable comparisons with existing research. Finally, Section 6 emphasizes the theoretical and practical implications, acknowledges the limitations of the study, and provides insightful suggestions for future research opportunities.

## 2. Theoretical Background and Hypotheses Development

### 2.1. Underpinning Theories 

#### 2.1.1. Stressor–Emotion Model

The stressor–emotion model offers a compelling psychological framework that illustrates how workplace stressors, such as interpersonal conflict, trigger emotional responses and lead to behaviors like deviant actions (Peng et al., 2023). Secured in the broader context of stress theory, this model underscores the vital importance of emotions in shaping how individuals react to stressful situations. Understanding this relationship can help organizations address these challenges and foster healthier workplace environments. The theory highlights the transactional process between individuals and their environment. 

The stressor–emotion model focuses on how stressors trigger emotional reactions resulting in unfit behaviors, particularly when the stressor is persistent or severe. Stressors are external or internal conditions that place demands on individuals, requiring them to adapt. Work stressors include challenges such as role ambiguity, workload, and interpersonal conflict (Dhal et al., 2022). According to the stressor–emotion model, how employees perceive and appraise these stressors influences their emotional responses. 

When employees experience interpersonal conflict, the difficulties evoked by these interactions are crucial in determining their subsequent behaviors (De Clercq & Belausteguigoitia, 2021). Workplaces threaten increased interpersonal deviance by enhancing emotional exhaustion and anger, as employees engage in behaviors such as gossiping, undermining colleagues, or reacting against others. These deviant behaviors typically express the emotional instability experienced because of unresolved conflict (De Clercq & Belausteguigoitia, 2021).

This research views workplace ostracism as both a result of inadequate coping strategies and an inherent stressor. Ostracized employees may feel excluded, and when employees feel ostracized by others, they may engage in retaliatory actions, such as deviant behavior. This is because workplace ostracism can create a cause in the external environment that reduces employees’ basic psychological needs (Fatima et al., 2023). Interpersonal conflict can trigger emotions in employees, which can lead to interpersonal deviance. It occurs when people with different values, opinions, or needs interact. It can be healthy and beneficial at times, but it can also become stressful and unproductive if not managed properly (Peng et al., 2023). The model demonstrates the crucial impact that emotions have on the correlation between conflict and behavior. 

This discussion explains why employees in conflict may engage in deviant behaviors. It underscores a vital area for organizational awareness and targeted intervention. Additionally, the model supports the investigation into workplace ostracism, which can be both a cause and a consequence of negative emotional responses to conflict. By incorporating emotional dynamics, this model provides a comprehensive framework for analyzing how stressors in the workplace, such as conflict and ostracism, lead to limitations in outcomes (Zaman et al., 2021).

#### 2.1.2. Conservation of Resources (COR) Theory

Stevan Hobfoll (1989) introduced the COR theory, a compelling psychological framework that highlights how individuals strive to acquire, maintain, and safeguard the resources they hold dear. These resources can be both tangible, like money and time, and intangible, such as social support and self-esteem. This theory underscores a fundamental human motivation: the desire to protect these resources from loss and, wherever possible, to enhance them. This theory asserts that stress fundamentally arises when people identify threats to their resources, encounter tangible resource losses, or fail to achieve their expected gains. Understanding this can help us to better navigate our reactions to stress and enhance our resilience (Ayub et al., 2021). 

In the workplace, resources may include social relationships, job stability, emotional support, and recognition from supervisors. When employees feel that their resources are under threat, they are likely to experience stress. According to Saepulloh and Laksana (2022), interpersonal conflict poses a threat to relational resources like trust and cooperation among colleagues.

Sarwar et al. (2020) describe workplace ostracism as a form of resource loss, depriving employees of essential social interactions, recognition, and support. According to the COR theory, employees who experience interpersonal conflict may perceive a threat to their social and emotional resources. Failing to replenish these vital resources increases the likelihood that individuals will turn to deviant behaviors as a means of self-protection or as retaliation against those they hold responsible for their losses.

#### 2.1.3. Conflict Expression (CE) Framework

The CE framework concentrates on the communication, interpretation, and management of conflicts in organizations (Mukhtar et al., 2020). How employees convey conflict shapes their perception by others, influencing interpersonal relationships and the overall dynamics of the organization. Direct and open expressions of conflict, while uncomfortable, can sometimes lead to resolution and improved understanding (Todorova et al., 2022). Effective conflict management involves recognizing the conflict, addressing the underlying issues, and facilitating open communication (Adham, 2023). 

This study highlights the importance of supervisors’ active, empathic listening in managing conflicts. By actively listening to employees and demonstrating empathy, supervisors can reduce the likelihood of conflict escalating into deviance and ostracism. When supervisors fail to acknowledge or address conflict, the risk of unresolved issues leading to deviant behavior increases (Jonsdottir & Kristinsson, 2020).

In the public sector, hierarchical structures and formal communication channels can sometimes exacerbate conflict by limiting open dialogue and discouraging employees from expressing their concerns directly. Interpersonal conflict can indirectly manifest through deviant behaviors or ostracism (Mazzei & Ravazzani, 2011). This framework elucidates how unresolved conflicts, especially those not publicly expressed, can trigger ostracism, subsequently leading to the escalation of deviant behavior. It highlights the importance of supervisors’ active, empathic listening as a tool for managing conflict. Managing the relationship between interpersonal conflict, deviant behavior, and ostracism involves cultivating a positive organizational environment. By addressing these dynamics, organizations can enhance collaboration and foster a culture of inclusivity and respect.

### 2.2. Interpersonal Conflict

Interpersonal conflict refers to a disagreement or clash between two or more individuals, often stemming from personal differences, perceptions, or values, while organizational conflict involves a dispute within a group or company structure. It typically arises from conflicting goals, policies, or resource allocation between different departments or teams within the organization. Essentially, interpersonal conflict is a smaller-scale disagreement between individuals, whereas organizational conflict is a broader issue impacting the dynamics of an entire group or company (Wogwu et al., 2023; Abugre, 2020). Administrative structures, resource constraints, and diverse workforce compositions can exacerbate interpersonal conflict in the public sector. When employees perceive that resources such as time, budget, or opportunities are limited, competition for these resources can escalate into conflict (Lemmetty et al., 2021). Research shows that individuals with high levels of ostracism engage in a diverse range of potential reactions in interpersonal conflict. These reactions include avoidance, aggression, withdrawal, emotional, and collaboration. They often depend on individual personality and the nature of the conflict. Understanding this can help us address and mitigate interpersonal tensions (Hetland et al., 2024). 

Moreover, communication barriers often contribute to conflict. Miscommunication or lack of clarity in job roles and expectations can lead to misunderstandings, which escalate into conflict. In a multicultural work environment, where employees might speak different languages or have different cultural expectations, these communication barriers can be a significant source of tension (Shaibu & Njoku, 2024). Organizational structures in the public sector often operate with slow decision-making and strict role definitions, leading to employee frustration and conflict (Nasir & Bashir, 2012). 

When employees engage in interpersonal deviance or experience reduced job satisfaction due to conflict, the quality of public service delivery suffers, affecting the citizens who rely on these services (Ingo, 2022). Addressing interpersonal conflict is therefore important not only for organizational efficiency, but also for maintaining the public’s trust in government institutions.

### 2.3. Interpersonal Deviance

Interpersonal deviance signifies intentional actions by employees that not only violate organizational standards but also jeopardize the well-being of their coworkers. Confronting and rectifying these behaviors promotes a healthier and more supportive workplace environment (Hua et al., 2023). These behaviors range from minor acts, like gossiping or spreading rumors, to more severe actions, such as verbal abuse, bullying, or intentionally sabotaging a colleague’s work (Nayak et al., 2022). In recent years, understanding interpersonal deviance has emerged as a critical component of organizational behavior research. It plays a significant role in shaping employee morale, enhancing productivity, and influencing the organization’s overall climate. Prioritizing these aspects is vital for improving a successful and positive work environment (Zahid & Nauman, 2024).

The stressor–emotion model (Spector & Fox, 2005) illustrates how interpersonal conflict serves as a significant stressor, evoking negative emotions like frustration and anger. These emotions can escalate into deviant behaviors that harm coworkers. Empirical evidence demonstrates that as conflict increases, the likelihood of employees engaging in disruptive actions against their colleagues rises in turn. Addressing interpersonal conflict is essential for fostering a healthier workplace environment (Singh et al., 2023). 

In the public sector, where employees often work under high levels of stress due to political pressures, resource constraints, and bureaucratic inefficiencies, the likelihood of interpersonal conflict and subsequent deviance may be higher (Akhmad et al., 2020). Additionally, employees often perceive the public sector as a stable and secure employment option, potentially leading to a sense of complacency or entitlement. Employees may engage in deviant behaviors as a form of protest or retaliation when expectations for career advancement or resource allocation fail to be met (Khattak et al., 2021). Moreover, administrative red tape limits public sector managers’ ability to swiftly address deviant behaviors, which can lead to their persistence and escalation.

### 2.4. Interpersonal Conflict and Interpersonal Deviance

Interpersonal conflict in the workplace is a critical focus in organizational behavior research, especially due to its powerful impact on shaping employee attitudes and influencing behaviors (Losada-Otalora et al., 2020). While some degree of conflict may be constructive in promoting diverse viewpoints and innovation, excessive or poorly managed interpersonal conflict can have detrimental effects on employees’ well-being and behavior, leading to undesirable outcomes, such as interpersonal deviance (Nadeem et al., 2020). Research indicates that interpersonal conflict is a significant predictor of interpersonal deviance. When employees experience conflict, they tend to respond with defensiveness or a desire for retaliation, which can undermine team dynamics and productivity. Perceived as the conflict’s source, these reactions typically escalate tensions and foster a toxic work environment. Addressing conflict proactively is essential for maintaining a healthy workplace (Anwar et al., 2020). Moreover, in the public sector, workplace conflict often arises due to factors like rigid bureaucratic structures, high-pressure workloads, limited resources, political influence, and diverse employee demographics. This conflict can be reinforced by supervisor behaviors, like inconsistent expectations, poor communication, lack of transparency, and favoritism, leading to a need for a systematic approach to managing these conflicts effectively. 

For instance, De Clercq et al. (2020) highlighted that interpersonal conflict often triggers emotional responses such as frustration, anger, and resentment; when employees experience negative emotions, they are more likely to engage in deviant behaviors and restore their sense of control. Additionally, deviant behavior can be caused by a variety of factors, including family issues, mental health concerns, peer pressure, societal influences, lack of clear social norms, personal personality traits, upbringing, life experiences, social pressures, and even genetic predisposition; essentially, a combination of individual and environmental factors can contribute to deviant behavior. This highlights the importance of addressing workplace stressors to promote healthier coping strategies.

The stressor–emotion model is particularly useful in explaining how interpersonal conflict leads to deviant behaviors (Spector & Fox, 2005). In a workplace setting, employees facing interpersonal conflict often experience feelings of alienation, frustration, or powerlessness. Intense emotions often drive individuals to resort to deviant behaviors as a means of releasing pent-up feelings or retaliating against their colleagues. Moreover, the COR theory underscores the importance of acquiring and safeguarding essential resources, such as time, energy, and social connections, which are critical for maintaining well-being and enhancing productivity. 

Prioritizing these resources is essential for fostering a supportive work environment where everyone can thrive. According to Hobfoll (1989) and De Clercq et al. (2020), it is crucial to prioritize these resources. When interpersonal conflict threatens these resources, such as by straining relationships or increasing stress, employees may resort to deviant behaviors to restore or compensate for their lost resources. The CE framework suggests that the expression of conflict, whether openly or covertly, can significantly influence the behavior of individuals involved (Park et al., 2020). 

Expressing conflicts in a hostile or passive-aggressive manner increases the likelihood of interpersonal deviance, as employees may respond in kind, either through confrontation or through more subtle forms of sabotage and ostracism. Conversely, constructive management of conflicts can mitigate the effect on employee behavior. Several mechanisms interconnect interpersonal conflict and interpersonal deviance (Jonsdottir & Kristinsson, 2020). Conflict also diminishes trust and cooperation, making it more likely for individuals to engage in behaviors that harm their colleagues, either as a form of retaliation or due to a breakdown in social cohesion (Engelbrecht, 2023). Building on the theoretical perspectives outlined above, the following hypothesis is proposed:

**H1:**  *Interpersonal conflict* *s are positively related to interpersonal deviance.*

### 2.5. Interpersonal Conflict and Workplace Ostracism

Interpersonal conflict can directly lead to workplace ostracism as unresolved tensions between individuals can cause colleagues to intentionally ignore someone, effectively pushing them to the margins of the social group within the workplace. Essentially, when conflicts escalate, people may resort to avoiding or isolating those involved as a coping mechanism. However, the effects of interpersonal conflict on employee behavior, well-being, and organizational outcomes can be equally, if not more, damaging (Singh et al., 2024). Scholars have explored the dynamics between interpersonal conflict and workplace ostracism, suggesting that interpersonal conflict may foster ostracism (Zahid et al., 2021). Conflict, when not effectively managed, can escalate and result in employees deliberately excluding their colleagues as a form of retaliation or avoidance. Ostracized employees may resort to deviant behaviors as a coping mechanism for the emotional stress resulting from social exclusion (Jiang et al., 2021). 

According to the COR theory, individuals aim not only to safeguard valuable resources, but also to nurture their social relationships, which are essential for overall well-being and resilience. When workplace ostracism threatens or disrupts these relationships, individuals often experience a significant reduction in their available resources. This loss can result in negative emotional responses and lead to counterproductive behaviors (Sarwar et al., 2020). While not all conflict leads to ostracism, unmanaged or persistent conflict can foster an environment where exclusion becomes a coping mechanism for those involved. Workers might turn to ostracizi9ng their peers to prevent additional conflict, alleviate interpersonal strain, or discipline individuals they consider troublesome (Gamian-Wilk & Madeja-Bien, 2021).

The stressor–emotion model (Spector & Fox, 2005) posits that emotions can impair employees’ ability to engage in healthy social interactions, leading them to withdraw from or exclude their colleagues. When employees are involved in conflict, they may choose to distance themselves from the source of the conflict by ignoring or excluding the individual, thereby engaging in workplace ostracism. Individuals involved in unresolved conflicts may deliberately exclude their colleagues to assert dominance or gain a sense of control over the situation (Adham, 2023). Conversely, constructive management of conflicts through open communication and empathy reduces the likelihood of ostracism, as employees are more likely to resolve their differences without resorting to social exclusion. Studies indicate that workers subjected to ostracism tend to withdraw from their tasks, diminish their dedication to the organization, and may even participate in counterproductive work behaviors (Yao et al., 2022). Based on the reviewed theoretical frameworks, this study formulates the following hypothesis:

**H2:**  *Interpersonal conflict is positively related to workplace ostracism.*

### 2.6. Workplace Ostracism and Interpersonal Deviance

Workplace ostracism occurs when individuals or groups intentionally ignore or exclude an employee, depriving them of essential social interactions and acceptance within the workplace environment (Gamian-Wilk & Madeja-Bien, 2021). Over time, this exclusion not only affects the emotional and psychological well-being of the ostracized employee but also fosters deviant behaviors that can damage the entire organization. In the workplace, ostracism can be divided into exclusion, social isolation, and silent treatment. Motivation strategies focus on fostering a sense of belonging, open communication, and individual support. Moreover, workplace ostracism is considered subjective because it is based on an individual’s perception of being excluded or ignored by others in the workplace. Interpersonal deviance, defined as behaviors aimed at harming coworkers or damaging interpersonal relationships, is one such consequence (Nadeem et al., 2020). The COR theory (Hobfoll, 1989) provides insights into the connection between ostracism and deviant actions. According to this theory, individuals seek to obtain, uphold, and safeguard important resources, including social ties, status, and mental health (Jabeen et al., 2022). Research consistently shows that employees who experience ostracism are significantly more likely to engage in a range of counterproductive work behaviors (CWB), such as interpersonal deviance. 

This connection highlights the critical need to foster an inclusive workplace environment. Shafique et al. (2020) view these behaviors as a direct response to the pain and rejection ostracism causes, as well as a form of retaliation against the organization or colleagues. According to the stressor–emotion model (Spector & Fox, 2005), workplace ostracism acts as a significant stressor, primarily triggering emotional responses such as anger, infuriation, and hurt, which in turn increase the likelihood of deviant actions.

Research has shown that employees facing social exclusion are more inclined to participate in harmful behaviors directed at their colleagues, as ostracism fosters feelings of bitterness and detachment from organizational norms and values (Yao et al., 2022). A study by (Jahanzeb et al., 2021) demonstrated that ostracized employees reported higher levels of interpersonal deviance, including gossiping, spreading rumors, and actively sabotaging their colleagues. Employees may resort to deviant behaviors to assert their existence or attract attention when they perceive a lack of appreciation or integration into the workplace’s social structure (Jahanzeb et al., 2021). These emotional reactions are consistent with the findings of Hua et al. (2023), who showed that workplace ostracism is a significant predictor of both interpersonal and organizational deviance. The theoretical foundations discussed in this section lead to the development of the following hypothesis:

**H3:**  *Workplace ostracism is positively related to interpersonal deviance.*

### 2.7. The Mediation Role of Workplace Ostracism

Gamian-Wilk and Madeja-Bien (2021) characterize workplace ostracism as the experience of colleagues socially sidelining or overlooking one another, leading to feelings of alienation and rejection. Research indicates that individuals who experience workplace ostracism often suffer from diminished psychological well-being, increased stress, and a heightened propensity to engage in interpersonal deviance (Jain et al., 2023). The stressor–emotion model posits that stressors, such as interpersonal conflict, elicit negative emotions that can influence subsequent behaviors (Spector & Fox, 2005). The COR theory, which emphasizes the importance of social resources in coping with stressors (Hobfoll, 1989), further supports the mediation effect of workplace ostracism. 

When employees face interpersonal conflict, they may perceive a threat to their social resources, leading to a sense of ostracism that exacerbates feelings of alienation. This social exclusion can reduce their ability to participate positively in the workplace, leading to the adoption of deviant behaviors as a coping mechanism for the perceived injustices in their social environment. Therefore, workplace ostracism not only occurs because of interpersonal conflict, but also reinforces a cycle of negativity that can permeate organizational culture (Liu et al., 2021).

The CE framework highlights that the expression of conflict can significantly vary based on individual characteristics and situational contexts. Factors such as organizational culture and the quality of relationships among employees play a crucial role in shaping these expressions. Understanding this can lead to more effective conflict resolution strategies (Todorova et al., 2022). Poor management of conflict can result in workplace ostracism, causing employees to retreat from interactions and reduce their engagement with their peers. This retreat may heighten emotions of frustration and detachment, leading employees to adopt interpersonal deviance to convey their discontent or regain a feeling of authority over their workplace (Ayub et al., 2021). Informed by the theoretical models examined, this research posits the following hypothesis:

**H4:**  *Workplace ostracism mediates the relationship between interpersonal conflict and interpersonal deviance*.

### 2.8. The Moderation Role of Supervisors’ Active Empathic Listening

Active, empathic listening by supervisors about workplace conflict should be proactive, neutral, and focused on facilitating open communication to effectively address issues, actively listen to all parties involved, and work towards a mutually agreeable solution, while ensuring fair treatment and upholding company policies, avoiding taking sides, or escalating the conflict unnecessarily (Teng et al., 2020). Empathic listening takes into account differences in age, gender, and experience. A listening style should be developed that involves understanding and responding to the concerns and perspectives of colleagues of different age groups, taking into account potential life experiences, communication preferences, and generational differences that may influence how they express themselves and perceive situations. This style should be employed to grasp employees’ emotions and perspectives on a situation, allowing the listener to respond with understanding and support, often by reflecting on their feelings and validating their experience, which can build trust and stronger working relationships. Women tend to naturally gravitate towards a more people-oriented listening style, focusing on emotions and understanding the speaker’s underlying feelings, while men may lean towards a more task-oriented style, prioritizing facts and problem-solving aspects of the conversation. However, these are generalizations, and individual variations exist across genders. In this study, it is posited that the existence of supervisors who engage in active listening and show empathy can significantly help to reduce the emotional strain associated with ostracism in in the public sector, through the practice of actively listening to citizens and stakeholders to truly understand their concerns, emotions, and perspectives, move beyond simply hearing their words, to build trust and foster better relationships with the community, ultimately leading to more effective policy-making and service delivery (Manusov et al., 2020). Supervisors who engage in active, empathic listening create an environment of trust and safety, allowing employees to express their feelings without fear of judgment. 

A nurturing atmosphere can reduce the impacts of workplace ostracism by cultivating a sense of belonging and affirmation among staff members. When employees view their supervisors as empathetic listeners, they are more prone to feeling supported and less likely to display interpersonal deviance as a reaction to ostracism (Ayub et al., 2021). The stressor–emotion model further elucidates the moderating effect of supervisors’ active empathic listening, positing that stressors such as workplace ostracism elicit negative emotional responses (Spector & Fox, 2005). Empathic listening serves as a protective mechanism against such adverse emotions by offering emotional assistance and affirming the feelings of employees (Kim & Leach, 2021). Consequently, employees who perceive their supervisors as engaging in active empathic listening may experience less emotional instability in response to ostracism, which can mitigate the likelihood of resorting to deviant behaviors as a coping strategy. Psychological distress potentially exists in the symbiotic connection between silence and ostracism, which exhibits a positive relationship (Zaman et al., 2021). 

Additionally, the COR theory emphasizes the significance of social resources in managing stress (Hobfoll, 1989). When supervisors actively listen and demonstrate empathy, they contribute to replenishing these social resources, helping employees navigate the emotional challenges posed by ostracism (Singh et al., 2024). This replenishment may diminish the positive connection between workplace ostracism and interpersonal deviance. Interpersonal conflict frequently leads to adverse emotions that can heighten conflicts among employees (Somaraju et al., 2022). When such conflicts are present, employees may feel inclined to retaliate or engage in deviant behaviors, particularly if they perceive a lack of supervisor support. 

Finally, research suggests that empathic listening can be beneficial across all levels of experience. It generally tends to function better with more experienced employees because they often have a greater understanding of workplace dynamics. Additionally, these employees better interpret nonverbal cues, and may be more receptive to open and honest communication, which are key elements of empathic listening. Additionally, a supervisor’s empathic listening can significantly improve negative emotions through employee morale, foster trust, encourage open communication, enhance problem-solving abilities, and ultimately lead to a more positive and productive work environment by making employees feel heard, understood, and valued, thus boosting their well-being and engagement with the company. However, supervisors who actively listen can create a mediating pathway that lessens the emotional fallout of conflict (De Clercq et al., 2020). By demonstrating understanding and support, they can reduce the likelihood that employees will turn to deviant behaviors as a means of expressing their frustrations. The preceding discussion of relevant theories supports the formulation of the following hypotheses:

**H5:**  *Supervisors’ active empathic listening moderately influences the positive correlation between workplace ostracism and interpersonal deviance, while this positive connection is less pronounced for employees whose supervisors exhibit high levels of empathic listening.*

**H6:**  *Supervisors’ active empathic listening moderately influences the positive correlation between interpersonal conflict and interpersonal deviance via workplace ostracism, indicating that this positive relationship is diminished for employees with supervisors who demonstrate strong empathic listening skills.*

**Figure 1:**
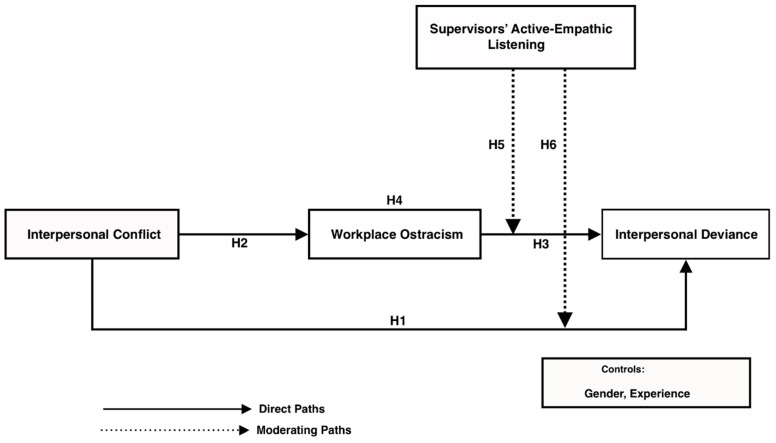
Conceptual model.

Figure 1 presents the conceptual framework of the study, illustrating the relationships between interpersonal conflict, workplace ostracism, and interpersonal deviance, with supervisors’ active-empathic listening moderating these effects. As depicted, interpersonal conflict directly influences workplace ostracism (H2), which in turn impacts interpersonal deviance (H3). Additionally, supervisors’ active-empathic listening moderates the relationship between workplace ostracism and interpersonal deviance (H6) as well as between interpersonal conflict and workplace ostracism (H5). Furthermore, gender and experience are included as control variables.

## 3. Research Methodology

### 3.1. Research Design, Sampling, and Data Collection Procedures

Research shows that interpersonal conflict within public sector organizations is notably different from that in private entities, highlighting the unique challenges and opportunities that exist in the public workforce (Prince et al., 2023). Deviant behaviors are significantly more dominant in the public segment than in private groups (Malik & Lenka, 2020). Therefore, this study’s participants were confined to employees from various public sector organizations in Jordan. This study employs the simple random sampling method to obtain samples, which is one of the most widely used sampling methods in social science studies (Iyiola & Rjoub, 2020). This method presents each member of the populace with a total prospect of remaining chosen. In line with the adopted sampling approach, this study obtained responses from employees with various profiles and experiences working in public organizations. Before data collection commenced, all participants were notified that their data would be kept confidential and utilized exclusively for academic research objectives. Voluntary participation was sought from the respondents after obtaining permission to conduct the survey from the official in charge.

This study utilized a two-wave research design, incorporating a one-month interval between the waves. This time gap was adequate to mitigate worries about reverse causality and was not lengthy enough to expect major organizational changes to happen during the data-gathering phase. The first wave administered questions related to demographic information, interpersonal conflict, and workplace ostracism. The second administered questions related to SAEL and interpersonal deviance. In sum, a total number of 759 questionnaires were administered offline and electronically, from which 552 responses were recovered. However, 51 responses were eliminated due to extremely highly repetitious and incomplete responses, and 501 complete responses were retained. Thus, a response rate of 66.01% was generated. 

Table 1 displays the demographic details of the participants. In terms of gender, 259 respondents (51.70%) identified as male, while 242 (48.30%) identified as female. Regarding the educational qualifications of the respondents, most of the respondents, 446 (89.02%), had at least a bachelor’s degree, showing that the majority had the necessary education to complete the survey. The ages of the respondents were as follows: below 25, 69 (13.77%), 25–34, 162 (32.34%), 35–44, 201 (40.12%), 45–54, 53 (10.53%), and above 55, 16 (3.19%). Finally, regarding their experience, most respondents, 432 individuals, or 86.23%, possessed at least six years of experience, suggesting that a significant portion of the participants had the varied experience necessary to complete the survey.

### 3.2. Measures

Interpersonal conflict was assessed using four items taken from (Spector & Jex, 1998). One example of an item is as follows: “How frequently do colleagues raise their voices at you in the workplace?”. Workplace ostracism was evaluated with ten items sourced from (Ferris et al., 2008). An example of one item is as follows: “Your greetings have been ignored in the work environment”. Supervisors’ active empathic listening was evaluated using eleven items modified from (Bodie, 2011). One of the original items stated, “I am aware of what others are not expressing”, which was changed to “My supervisor demonstrates attentiveness through body language”. Interpersonal deviance was assessed using seven items sourced from (Bennett & Robinson, 2000). One item example is as follows: “Embarrassed someone publicly in a work setting”. Detailed measurement items for all constructs are presented in Appendix A, Table A1.

### 3.3. Analytical Procedures

Initially, descriptive statistics, including mean, SD, and correlations among variables were calculated with SPSS 27. Subsequently, CFA was conducted to assess the reliability and validity of the survey instruments utilizing AMOS 24. The relationships direct, indirect, conditional direct, and conditional indirect (as depicted in Figure 1) were evaluated through the PROCESS macro (Models 4 and 15) (Hayes, 2017). The bootstrapping procedure with a 95% confidence interval (CI) (i.e., 5000 resamples) was used to test the indirect effect, the conditional direct, and indirect effects. Where the 95% CI excludes zero, a statistical significance is observed. The significance of the interaction term between interpersonal conflict and supervisors’ active empathic listening for interpersonal deviance was used to examine the moderating effect. Additionally, a straightforward slope analysis evaluated the conditional indirect impact of supervisors’ active empathic listening on how interpersonal conflict affects interpersonal deviance via workplace ostracism. Furthermore, the bootstrap confidence interval was utilized to evaluate the significance of the moderated mediation index. In the moderation assessment, both experience and gender were included as covariates.

### 3.4. Non-Response Bias

To identify potential non-response bias, we followed the approach outlined in (Armstrong & Overton, 1977) to analyze the demographic traits (such as age variations) between early and late respondents. As a result, we split the final sample into two groups: the initial half of the respondents comprised the early group, while the latter half represented the late group. The ages of respondents showed no significant difference between the analyses of early and late respondents, with a t-value of 0.413 and a *p*-value of 0.602. Therefore, non-response bias was not considered a major issue in this study.

## 4. Analysis and Results

### 4.1. Common Method Bias (CMB)

Survey-based studies may be prone to CMB, especially when single respondents rate both the independent and dependent variables (Podsakoff et al., 2003). Therefore, we adhered to various techniques proposed by (Podsakoff et al., 2003) as remedies, including procedural and statistical measures for CMB. First, in the survey design, we arranged the questions related to the dependent variables at the beginning of the questionnaire and ensured that they were physically distanced from those related to independent variables. In addition, midpoint labels were included in the scales. An introductory statement was included in the questionnaire to guarantee the complete anonymity of the respondents. Furthermore, the survey participants were the suitable key informants for the sample obtained, as each respondent was a full-time employee of the public organization they represented. 

In addition, regarding the statistical metrics, this research utilized Harman’s first factor test to assess the possible impact of CMB. All the items were included in an exploratory factor analysis. The results indicate that the first factor explains 21.99% of the total variance, which is below the suggested threshold of 50%. This research also employs the marker variable strategy (Lindell & Whitney, 2001). We incorporated a theoretically unrelated construct into the survey and analyzed the relationship between the marker variable and the primary variables of the study. The findings reveal that the marker variable exhibited low correlations (below 0.05) with the primary variables. Therefore, both the procedural and the statistical measures suggest that no significant CMB was detected in the present research.

### 4.2. Measurement Model: Reliability and Validity of the Survey Instruments

**Figure 2:**
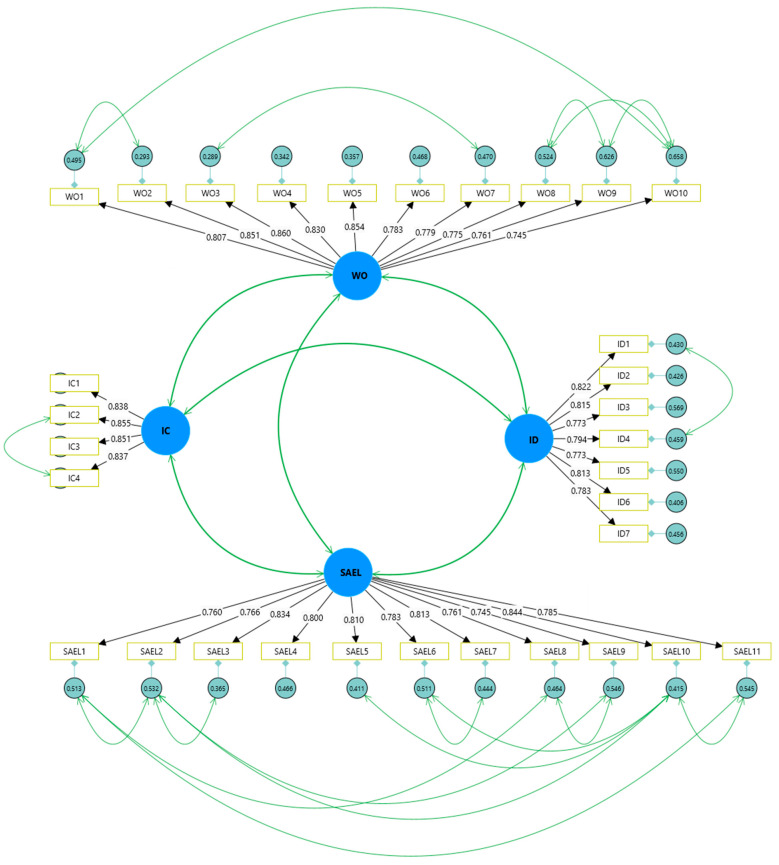
CFA results showing factor loadings.

The reliability of the measurement variables was assessed through Cronbach’s alpha. Table 2 displays the findings from the measurement model. The Cronbach’s alpha coefficients for all the constructs ranged from 0.886 to 0.942, indicating that these constructs possess suitable reliability (Nunnally, 1978). Subsequently, we conducted a CFA to evaluate the convergent and discriminant validity of the constructs. All the measurement variables demonstrated significant loading on their corresponding constructs (*p* < 0.001), with all the factor loadings exceeding 0.7 (ranging from 0.745 to 0.860), which is depicted in Figure 2. The composite reliability (CR) values ranged from 0.909 to 0.949, while the average variance extracted (AVE) values fell between 0.627 and 0.715, both surpassing the thresholds of 0.7 and 0.5, respectively. All of these metrics sufficiently meet the criteria for convergent validity, suggesting that the survey tools are dependable and coherent. Furthermore, the square root of each AVE was shown to exceed the adjacent correlations (as illustrated in Table 3), thus providing robust evidence for discriminant validity (Fornell & Larcker, 1981).

Finally, our comprehensive theoretical model (baseline model) demonstrated a strong fit with the collected data (Bagozzi & Yi, 1988), with indices such as CMIN/df = 2.912 (less than 3), GFI = 0.855 (greater than 0.8), CFI = 0.936 (greater than 0.9), IFI = 0.936 (greater than 0.9), NFI = 0.910 (greater than 0.9), TLI = 0.926 (greater than 0.9), and RMSEA = 0.61 (less than 0.08). The findings regarding the overall fit of the proposed model are presented in Table 2.

### 4.3. Testing Direct and Indirect (Mediating) Path Hypotheses

We examined the direct and indirect relationships using PROCESS macro model number 4 and reported the results in Table 4. The findings provide evidence supporting H1, which posits that interpersonal conflict is positively linked to interpersonal deviance. According to Table 4, interpersonal deviance shows a positive association with interpersonal conflict (β = 0.409, t = 11.129, *p* < 0.001). H2 predicted a strong connection between interpersonal conflict and workplace ostracism, with the results indicating a positive relationship (β = 0.856, t = 35.787, *p* < 0.001). H3 suggested a positive link between workplace ostracism and interpersonal deviance, which was confirmed (β = 0.542, t = 14.782, *p* < 0.001). Consequently, H1 through H3 were all validated by the study’s outcomes.

Following Baron and Kenny (1986)’s mediation approach, at the inclusion of workplace ostracism as the mediator, the main (i.e., direct effect) between interpersonal conflict and interpersonal deviance remained significant. Based on this result, partial mediation was observed. 

Furthermore, to ensure the most reliable results for the mediation, we utilized bootstrapping of 5000 resamples at 95% CI to determine the confidence intervals to explore the mediation effect of workplace ostracism. Table 4 demonstrates that workplace ostracism partially mediates the correlation between interpersonal conflict and interpersonal deviance, as the confidence interval from the bootstrapping results does not include zero (β = 0.464, SE = 0.043, 95% CI [0.379, 0.546]). Therefore, we found support for H4.

### 4.4. Conditional Direct and the Moderated Mediated Path

**Figure 3:**
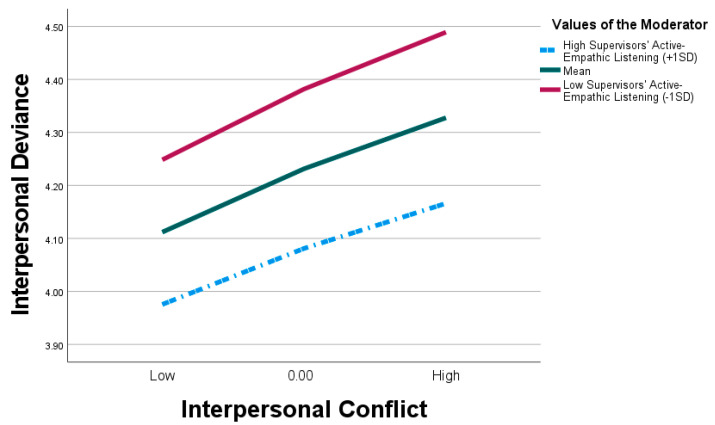
Interaction effect (IC*SAEL) on interpersonal deviance.

Using the PROCESS macro model 15, this study examined the conditional direct and moderated mediated paths. In the analyses, both experience and gender were included as covariates, and the constructs were mean-centered to mitigate multicollinearity concerns. The same criteria for establishing significance required a ‘non-zero’ value within the upper and lower limits of the 95% confidence interval (Hayes, 2017). In Model 2 in Table 5, interpersonal conflict predicts interpersonal deviance (β = 0.129, t = 2.561, *p* < 0.01), with the relationship being moderated by the interaction between interpersonal conflict and supervisors’ active empathic listening (β = −0.119, SE = 0.057, t = −2.108, 95% CI [−0.230, −0.008]). To further understand the moderating path (i.e., the conditional direct effect), we computed the moderating path at three levels: −1SD (below the sample mean), the mean, and +1SD (above the sample mean). Employing the simple slope test (Aiken, 1991), the three levels represent low, medium, and high groups, respectively. The results are presented in Table 5 and Figure 3; the direct relationship between interpersonal conflict and interpersonal deviance depends on the levels of supervisors’ active empathic listening. The positive relationship between interpersonal conflict and interpersonal deviance decreases for employees who perceived high levels (β = 0.283, SE = 0.063, t = 4.135, 95% CI [0.149, 0.418]) of SAEL, and the relationship is stronger for employees who perceived low levels (β = 0.503, SE = 0.063, t = 7.943, 95% CI [0.378, 0.627]) of SAEL. Thus, we found support for H5. 

**Figure 4:**
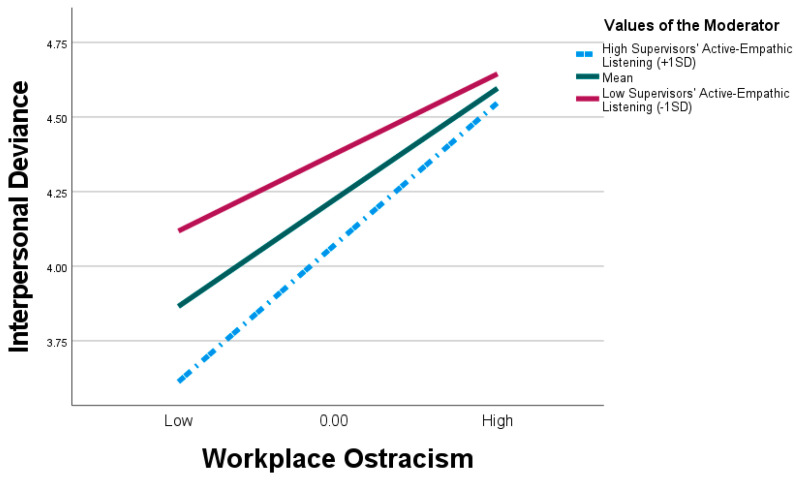
Supervisors’ active empathic listening moderates the indirect relationship between interpersonal conflict and interpersonal deviance through workplace ostracism.

Furthermore, using the bias-corrected bootstrap, this study examined the conditional indirect effect of supervisors’ active empathic listening. The significance of the indirect relationship between interpersonal conflict and interpersonal deviance through workplace ostracism was examined at three different levels of the moderator (SAEL). The three conditions denote the lower, mean, and high levels of the moderator at −1SD, 0, and +1SD, respectively. Table 5 and Figure 4 illustrate that the indirect influence of workplace ostracism on the correlation between interpersonal deviance and interpersonal deviance diminishes (i.e., becomes less significant) at high levels of supervisors’ active empathic listening (β = 0.242, SE = 0.071, 95% CI [0.111, 0.386]). Conversely, this relationship is more robust at low levels of supervisors’ active empathic listening (β = 0.430, SE = 0.067, 95% CI [0.295, 0.555]). The results indicate that for higher levels of supervisors’ active empathic listening, interpersonal conflict has a lesser indirect impact on interpersonal deviance through workplace ostracism. The creation of a moderated mediation model was validated by the moderated mediation index, as the confidence interval did not include zero, showing 0.102 (95% CI = [0.015, 0.211]). Therefore, we found support for H6.

## 5. Discussion

This research provides valuable insights into the interplay between interpersonal conflict, workplace ostracism, and employee deviance in the public sector, with a particular emphasis on the moderating role of supervisors’ active empathic listening. By integrating the stressor–emotion model, the COR theory, and the CE framework, this research extends existing knowledge about how interpersonal conflict impacts employee behavior and how specific organizational factors, such as leadership listening, can mitigate negative outcomes.

This research established that interpersonal conflict positively correlates with interpersonal deviance. The results align with prior research emphasizing the adverse effects of workplace conflicts on employee behavior. Researchers like Zahid and Nauman (2024) identified workplace conflict as a key predictor of negative behaviors, including incivility and aggression. This study adds to the existing literature by confirming the existence of this relationship within the public sector, where organizational frameworks and bureaucratic demands can heighten the emotional stress associated with interpersonal conflict.

The significant positive correlation between interpersonal conflict and workplace ostracism is consistent with earlier studies indicating that conflict frequently results in social exclusion within organizations. Research (Zaman et al., 2021) similarly found that conflict often leads to ostracism, as organizations aim to minimize disruptions by isolating those involved in disputes. This research contributes to the existing literature by empirically showing the robustness of this relationship within the public sector, where hierarchical systems and formal communication pathways can intensify the effects of social exclusion.

The finding that workplace ostracism has a positive relationship with interpersonal deviance underscores the importance of ostracism in comprehending employee misconduct. This outcome aligns with prior research by Ayub et al. (2021), which indicated that employees who feel ostracized often resort to retaliatory actions to cope. This study advances this aspect of the literature by showing that workplace ostracism is a significant mediator between interpersonal conflict and deviance, offering empirical support for its role in translating conflict into negative behavioral outcomes.

The partial mediation of workplace ostracism in the link between interpersonal conflict and interpersonal deviance highlights the critical role of social exclusion in influencing how employees react to conflict. This finding extends the work of previous researchers, such as (Zahid & Nauman, 2024), by providing a view of how conflict escalates into deviance. This study adds to this by demonstrating that workplace ostracism can be a crucial intermediary in this process, indicating that conflict is more likely to result in deviance when it is met with social exclusion rather than constructive resolution.

A key contribution of this research is the discovery that supervisors’ active empathic listening moderates the connection between workplace ostracism and interpersonal deviance. This finding underscores the significance of empathetic leadership in reducing the harmful consequences of workplace conflict and social exclusion. Earlier research by (Singh et al., 2024) has indicated that leadership is vital in resolving conflicts, but this study specifically demonstrates how empathic listening can lower the likelihood of ostracized employees engaging in deviant actions. By confirming the moderating influence of empathic listening, this study contributes to the expanding literature on emotional intelligence’s role in leadership and conflict resolution. Additionally, this research shows that supervisors’ active empathic listening diminishes the indirect impact of interpersonal conflict on interpersonal deviance via workplace ostracism. This suggests that higher levels of empathic listening can mitigate the progression of conflict into deviance by decreasing employee social exclusion. Recent research in the public sector regarding workplace ostracism highlights the crucial role of inclusive leadership practices, clear anti-ostracism policies, and accessible reporting mechanisms in raising awareness and mitigating the negative impacts of social exclusion on employee behavior and wellbeing (Chen, 2024). Empathic listening enables the expression of conflict in a way that fosters understanding and resolution, rather than exclusion and deviance (Bodie, 2011).

## 6. Conclusions

### 6.1. Theoretical Contribution

This research provides several key theoretical advancements in comprehending interpersonal conflict, workplace ostracism, and interpersonal deviance. By integrating the stressor–emotion model, COR theory, and the CE framework, this research adds innovative perceptions to the variables.

The stressor–emotion model posits that stressors in the workplace, such as interpersonal conflict, trigger negative emotional responses that can lead to maladaptive behaviors like deviance (Spector & Fox, 2005). This research expands the application of this model by providing empirical evidence that interpersonal conflict not only acts as a stressor, leading to negative emotional states, but also plays a crucial role in predicting interpersonal deviance. Additionally, this study enhances the stressor–emotion model by emphasizing the importance of workplace ostracism as a mediating element. Workplace ostracism not only intensifies negative emotional responses, but also serves as a mechanism through which conflict escalates into deviant behaviors. This mediating effect enhances the stressor–emotion model by incorporating a social aspect into the stressor–emotion–behavior sequence. This addition shows how conflicts can lead to adverse employee actions.

Workplace ostracism can negatively affect employee well-being and the stressor–emotional model. Meanwhile, it is seen as a stressor that depletes employees’ emotional resources and negatively affects their attitudes and behaviors (Bedi, 2021). This has profound implications for understanding workplace ostracism. Furthermore, this study verifies the observation that when employees perceive ostracism, there is a loss of psychological resources, which causes emotional exhaustion (Xing et al., 2021), and enriches the research on negative workplace behaviors and wellbeing (Wang et al., 2023).

This research offers a notable theoretical advancement to the COR theory by demonstrating how interpersonal conflict in the workplace diminishes employees’ emotional resources, resulting in negative consequences like ostracism and deviant behavior (Hobfoll, 1989). The COR theory traditionally focuses on how resource depletion leads to stress and maladaptive coping mechanisms, but this research highlights that ostracism, as a form of social resource scarcity, determines negative behaviors, like deviance. Ostracized individuals, denied social and emotional support, may resort to deviant behaviors to cope with their loss of resources or as a disciplinary mechanism. This finding enhances the COR theory by highlighting the social implications of resource depletion, especially regarding workplace relationships and conflict.

The CE framework posits that the expression of conflict within organizations can shape its outcomes, with constructive expression leading to resolution and destructive expression leading to further escalation (Todorova et al., 2022). This study advances this framework by showing that workplace ostracism serves as a destructive form of conflict expression, exacerbating the negative effects of interpersonal conflict. By listening actively and empathetically to employees involved in conflict or those experiencing ostracism, supervisors can prevent the escalation of conflict into deviance. Empathetic listening reduces the indirect impact of interpersonal conflict on interpersonal deviance in the workplace, highlighting the critical role of leadership actions in shaping conflict management and perception within organizations. By demonstrating that supervisors who engage in active empathic listening can reduce the negative effects of ostracism and conflict, this study offers a new theoretical pathway for understanding how leadership behaviors can buffer the emotional and social costs of workplace conflict. This contributes to the existing body of research on leadership by highlighting the significance of empathy as an essential leadership skill in handling interpersonal conflict and fostering a more inclusive and nurturing workplace.

### 6.2. Practical and Managerial Implications

The findings of this research on interpersonal conflict, workplace ostracism, and interpersonal deviance provide valuable perceptions with important practical and managerial implications, particularly for public sector organizations in Jordan.

A significant finding of this research is the positive correlation between interpersonal conflict and interpersonal deviance. Management and HR teams should prioritize conflict resolution training initiatives that empower employees and supervisors with the ability to handle disputes constructively. These programs could include mediation techniques, communication skills workshops, and collaborative problem-solving strategies, which help employees express and resolve conflicts in a healthy, productive manner (Irawan, 2024). This study revealed that workplace ostracism acts as a mediator in the correlation between interpersonal conflict and interpersonal deviance. This suggests that employees experiencing conflict are more vulnerable to peer exclusion, which in turn increases the likelihood of engaging in deviant behavior (Shafique et al., 2020). Organizations should foster a culture of inclusion by encouraging teamwork and collaboration across all levels of the hierarchy. Government organizations can facilitate team-building events, interdepartmental initiatives, and mentorship programs to foster a sense of community and social unity among their staff. Additionally, HR departments should regularly monitor employee interactions and provide anonymous feedback mechanisms, such as employee surveys, to identify instances of ostracism (Wang et al., 2023). To prevent the escalation of deviance, HR departments should take immediate action to support ostracized employees through conflict mediation or counseling (Mujtaba & Senathip, 2020).

One of the key findings of this research is the impact of supervisors’ active empathic listening on the relationship between workplace ostracism and interpersonal deviance. Organizations should prioritize the development of empathetic leadership skills through targeted training programs. These programs can focus on active listening techniques, emotional intelligence, and conflict resolution skills (Jonsdottir & Kristinsson, 2020). We should train supervisors to identify signs of ostracism and interpersonal conflict and to respond with understanding, support, and empathy. Moreover, promoting empathetic leadership can improve overall employee engagement and retention. Staff members tend to experience greater job satisfaction and organizational commitment when they perceive that their supervisors acknowledge and appreciate their issues (Negoro & Wibowo, 2021). This aspect is especially crucial in public sector environments, where bureaucratic procedures and hierarchical frameworks can occasionally lead to a feeling of alienation between employees and leadership. Compassionate leadership addresses this disconnect by promoting transparent communication and a nurturing workplace atmosphere. By adopting these approaches, public sector organizations can boost employee wellness, enhance organizational effectiveness, and establish a more equitable and efficient work setting (Ramachandran et al., 2024).

Since workplace ostracism affects employee wellbeing, managers should minimize or avoid ostracism in the workplace. For example, companies can reduce the incidence of ostracism by encouraging employees to use face-to-face communication, strengthening care for employees through multiple methods, encouraging employees to participate in social activities, increasing communication opportunities between colleagues, improving emotional communication, promoting mutual understanding, and reducing the possibility of ostracism.

In addition, workplace ostracism consumes employees’ emotional resources and causes emotional exhaustion. Organizations should provide employees with more organizational support, such as a good communication environment and a cordial atmosphere, which makes employees develop positive emotions toward work and helps them regain resources to overcome the negative effects of ostracism. At the same time, organizations can establish a psychological counseling mechanism for employees to reduce their level of emotional exhaustion and provide opportunities to replenish resources in an attempt to eliminate negative emotions.

### 6.3. Limitations and Future Studies

Although this research offers a significant understanding of the interactions between interpersonal conflict, workplace ostracism, and interpersonal deviance within public sector entities, it is important to recognize certain limitations. This research took place in Jordan’s public sector. Although the results have significant relevance for public sector institutions in these areas, their applicability to different sectors or nations may be restricted. To improve the applicability of these findings, subsequent studies should investigate the functioning of interpersonal conflict, workplace ostracism, and interpersonal deviance in other fields, including private and non-profit organizations. This research utilized a cross-sectional framework, gathering data at one specific moment. Although this method facilitates the analysis of correlations among variables, it restricts the capacity to draw conclusive causal connections. Subsequent research should implement longitudinal methodologies to investigate how the connections between interpersonal conflict, workplace ostracism, and deviance develop over time. Utilizing self-reported data introduces possible concerns regarding common method bias and social desirability bias. The respondents might have intentionally or unintentionally answered in a manner that conformed to socially acceptable standards instead of accurately portraying their genuine behaviors or emotions. Although this research focused on workplace ostracism and the active empathic listening of supervisors, upcoming studies should explore additional mediating and moderating elements that may influence the relationships analyzed.

## Figures and Tables

**Table 1:** Sample’s demographics.

Characteristics (n = 501)	Classification	Frequency	Proportion (%)
Gender			
	Male	259	51.70
	Female	242	48.30
Education			
	Below graduate degree	55	10.98
	Graduate degree	304	60.68
	Above graduate degree	142	28.34
Age			
	Below 25	69	13.77
	25–34	162	32.34
	35–44	201	40.12
	45–54	53	10.58
	Above 55	16	3.19
Experience			
	Less than 5 years	69	13.77
	Between 6 and 10	188	37.52
	Between 11 and 15	219	43.72
	15 or more	25	4.99

**Table 2:** Reliability and validity of the scales.

Construct	Codes	Factor Loading	Cronbach’s Alpha	CR	AVE
IC			0.942	0.909	0.715
	IC1	0.838			
	IC2	0.855			
	IC3	0.851			
	IC4	0.837			
WO			0.891	0.949	0.649
	WO1	0.807			
	WO2	0.851			
	WO3	0.860			
	WO4	0.830			
	WO5	0.854			
	WO6	0.783			
	WO7	0.779			
	WO8	0.775			
	WO9	0.761			
	WO10	0.745			
SAEL			0.886	0.949	0.627
	SAEL1	0.760			
	SAEL2	0.766			
	SAEL3	0.834			
	SAEL4	0.800			
	SAEL5	0.810			
	SAEL6	0.783			
	SAEL7	0.813			
	SAEL8	0.761			
	SAEL9	0.745			
	SAEL10	0.844			
	SAEL11	0.785			
ID			0.925	0.924	0.634
	ID1	0.822			
	ID2	0.815			
	ID3	0.773			
	ID4	0.794			
	ID5	0.773			
	ID6	0.813			
	ID7	0.783			
CMIN/df = 2.912, GFI = 0.855, CFI = 0.936, >0.9, IFI = 0.936, NFI = 0.910, TLI = 0.926, and RMSEA = 0.61					

Note: Interpersonal Conflict = IC, Workplace Ostracism = WO, Supervisors’ Active Empathic Listening = SAEL, Interpersonal Deviance = ID.

**Table 3:** Descriptive statistics, intercorrelations, and discriminant validity.

Construct	Mean	Standard Deviation	IC	WO	SAEL	ID	Experience	Gender
IC	3.490	1.048	(0.845)					
WO	3.808	0.819	0.618 **	(0.805)				
SAEL	3.654	0.793	0.481 **	0.549 **	(0.792)			
ID	3.721	0.998	0.566 **	0.516 **	0.536 **	(0.796)		
Experience	1.53	0.622	0.041	0.017	0.005	0.015	-	
Gender	3.105	1.277	−0.057	−0.002	−0.009	−0.026	−0.012	-

Note: values in diagonal parentheses are square roots of AVEs, ** *p* < 0.01.

**Table 4:** Mediation analysis (PROCESS macro model number 4).

Direct Effects (Using Bootstrap Bias-Corrected Technique CI 95%)						
	Hypothesized Relationships	Coeff	S. E	t	LL	UL
H1	IC → ID	0.409	0.037	11.129 ***	0.337	0.481
H2	IC → WO	0.856	0.024	35.787 ***	0.809	0.903
H3	WO → ID	0.542	0.037	14.782 ***	0.470	0.614
R^2^ =						
	Direct effect of X on Y	0.409	0.037	11.119 ***	0.337	0.481
	Total effect of X on Y	0.873	0.023	37.784 ***	0.828	0.919
Indirect effects Indirect using the bootstrapping methodEffect BootSEBootLLBootUL						
H4	IC → WO →	0.464	0.043		0.379	0.546

Note: S.E = standard error, LL = lower level of confidence interval, UL = upper level of confidence interval, *** = *p* < 0.001.

**Table 5:** Moderated mediation analysis (PROCESS macro model number 15).

Hypothesized Relationships	Coeff.	S. E	t	95% Confidence Interval Lower Level Upper Level	
Model 1:					
Covariate: Experience → WO	0.027	0.031	0.046 ^ns^	−0.059	0.009
Covariate: Gender → WO	0.021	0.020	0.030 ^ns^	−0.049	0.007
IC→ WO	0.856	0.024	35.787 ***	0.809	0.903
Model 2:					
Covariate: Experience → WO	0.039	0.018	0.041 ^ns^	−0.039	0.011
Covariate: Gender → WO	0.043	0.030	0.049 ^ns^	−0.034	0.006
IC → ID	0.129	0.050	2.561 **	0.030	0.227
WO → ID	0.393	0.040	9.726 ***	0.314	0.472
SAEL → ID	0.163	0.036	4.478 ***	0.092	0.235
H5: IC*SAEL → ID	−0.119	0.057	−2.108 *	−0.230	−0.008
Workplace*SAEL → ID	0.016	0.052	0.313ns	−0.086	0.118
The specific conditional values of SAEL for the relationship between IC and ID					
−1SD (below the mean)	0.503	0.063	7.943 ***	0.378	0.627
Mean	0.393	0.040	9.726 ***	0.314	0.472
+1SD (above the mean)	0.283	0.069	4.135 ***	0.149	0.418
H6: The conditional indirect effect of X on Y: The conditional indirect effect of IC on ID through WO at different levels of SAEL on the relationship					
−1SD (below the mean)	0.430	0.067		0.295	0.555
Mean	0.336	0.044		0.251	0.426
+1SD (above the mean)	0.242	0.071		0.111	0.386
Index of moderated mediation by SAEL					
	Index	BootSE		BootLLCI	BootULCI
IC→ WO → ID	0.102	0.058		0.015	0.211

Note: ULCI = lower level of confidence interval, ULCI = upper level of confidence interval, * = *p* < 0.05, ** = *p* < 0.01, *** = *p* < 0.001, ns = not significant.

## Data Availability

The data from this study can be requested from the conservation of resources theory’s corresponding author, Ahmad Alzubi.

## Appendix A

**Table A1:** Measurement items.

Interpersonal conflict (Spector & Jex, 1998)
1. How often do you get into arguments with others at work
2. How often do other people yell at you at work
3. How often are people rude to you at work
4. How often do other people do nasty things to you at work
Workplace ostracism (Ferris et al., 2008)
1. Others ignored you at work
2. Others left the area when you entered
3. Your greetings have gone unanswered at work.
4. You involuntarily sat alone in a crowded lunchroom at work
5. Others avoided you at work
6. You noticed others would not look at you at work.
7. Others at work shut you out of the conversation
8. Others refused to talk to you at work
9. Others at work treated you as if you weren’t there
10. Others at work did not invite you or ask you if you wanted anything when they went out for a coffee break.
Supervisor active empathic listening, adapted from (Bodie, 2011)
1. My supervisor assures me that he/she is listening by using body language
2. My supervisor assures me that he/she is aware of what others imply but do not say
3. My supervisor keeps track of points others make
4. My supervisor summarizes points of agreement and disagreement when appropriate
5. My supervisor assures me that he/she will remember what others say
6. My supervisor assures me that he/she listens more than just the spoken words
7. My supervisor assures me that he/she is listening by using verbal acknowledgement
8. My supervisor assures me that he/she is receptive to different ideas
9. My supervisor asks questions that show understanding of others positions
10. My supervisor understands how others feel
11. My supervisor is sensitive to what others are not saying
Interpersonal deviance (Bennett & Robinson, 2000)
1. Made fun of someone at work
2. Said something hurtful to someone at work
3. Made an ethnic, religious, or racial remark at work
4. Cursed at someone at work
5. Played a mean prank on someone at work
6. Acted rudely toward someone at work
7. Publicly embarrassed someone at work
